# Efficacy and Safety of Low-Dose Everolimus as Maintenance Immunosuppression in Cardiac Transplant Recipients

**DOI:** 10.1155/2012/976921

**Published:** 2012-04-17

**Authors:** Uwe Fuchs, Armin Zittermann, Uwe Schulz, Jan F. Gummert

**Affiliations:** Clinic for Thoracic and Cardiovascular Surgery, Heart and Diabetes Center North Rhine-Westphalia, Ruhr University Bochum, Georgstraße 11, 32545 Bad Oeynhausen, Germany

## Abstract

For cardiac transplant (CTx) recipients, the recommended everolimus (EVL) dose is 0.75 mg bid or 1.5 mg bid and the target trough blood level is 3–8 *μ*g/L. We studied a cohort of 56 CTx patients with chronic kidney disease receiving 0.75 mg bid EVL to maintain blood levels of 5–8 ug/L (designated RD group) and a cohort of 51 CTx patients with chronic kidney disease receiving 0.5 mg bid to maintain blood levels of 3–5 ug/L (designated LD group). The primary endpoint was a composite of death, rejection and premature EVL discontinuation up to 1 year after introduction of EVL. The primary endpoint was reached by 32% of patients in the LD group and by 41.1% of patients in the RD group (*P* = 0.361). Biochemical safety parameters were comparable in both groups. Our results indicate that low-dose EVL may be as effective and safe as regular dose EVL.

## 1. Introduction

The use of calcineurin inhibitors (CNIs) such as cyclosporine (CSA) and tacrolimus (TAC) has dramatically increased medium-term life expectancy after heart transplantation but has had only limited impact on long-term outcomes for heart transplant recipients [1]. One of the most important predictors for patient mortality at >5 years after heart transplantation is cardiac allograft vasculopathy (CAV), which accounts for 31% of deaths. Neither cyclosporine nor tacrolimus has been shown to prevent the development of CAV [1]. CNI administration also increases the risk of chronic kidney disease (CKD) [2]. Between 3% and 10% of cardiac transplant recipients will ultimately develop CKD stage 5 [3].

The mammalian target of rapamycin (TOR) inhibitor everolimus (EVL) potentially reduces CAV, while maintaining the low cellular rejection rates seen with standard therapy [4]. In addition, EVL allows a marked reduction of CSA exposure in de novo cardiac transplant recipients, subsequently leading to an early protection of renal function [5]. As medical care for cardiac transplant recipients, in combination with low-dose CSA, EVL is able to achieve a stable kidney function [6].

The EVL dosage is according to trough blood levels 0.75 or 1.5 mg twice daily. Target blood levels are 3 to 8 *μ*g/L, with 6 to 8 *μ*g/L considered as the optimal range for most patients [7]. However, tolerability and safety of EVL therapy remain a concern with medical conditions such as pneumonitis, effusions, mouth ulcers, edema, and impaired wound healing [4]. Approximately 5% of cardiac transplant recipients develop potentially life-threatening lingual edema under EVL therapy [8]. Further, EVL leads to hyperlipidemia and dyslipidemia [9, 10].

It is currently not known whether patients would benefit from a low EVL dose. It was therefore the aim of this cohort study to compare efficacy and safety of low-dose EVL with regular dose administration in cardiac transplant recipients.

## 2. Materials and Methods

### 2.1. Patients

**Figure 1:**
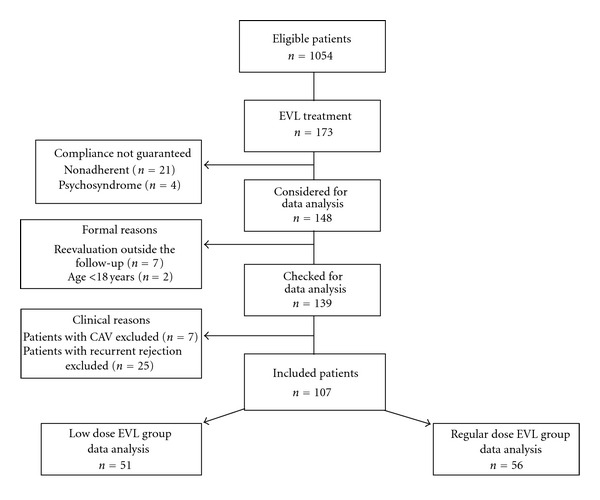
Study flow chart.

This investigation is based on patients who were changed from immunosuppressive therapy with CNI, purine antagonist, and methylprednisolone to EVL plus low-dose CNI therapy at our clinic between January 2004 and June 2006. Of 1054 eligible cardiac transplant recipients, 173 received EVL plus low-dose CNI therapy (Figure 1). After exclusion of 21 patients known to be nonadherent with the immunosuppressive medication and four patients with psychosyndrome, 148 patients were checked. Seven patients had less than 2 visits per year and two patients were younger than 18 years of age. Seven other patients were already diagnosed as having CAV and 25 patients suffered already from recurrent rejection before conversion to EVL, for example, more than 3 rejections since their cardiac transplantation indicating insufficient immunosuppression. Thus, 107 patients could be included into our data analysis. Inclusion criteria for conversion to EVL were a serum creatinine concentration >1.6 mg/dL (*n* = 104), neurotoxicity of CNI inhibitors (*n* = 2), and a tumour diagnosis (*n* = 1). Of the 107 patients, a cohort of 56 patients received a regular EVL dose (0.75 mg bid, designated RD group) to maintain blood levels to 5–8 ug/L. The RD group was switched over to EVL between January 2004 and December 2004. Due to concerns about potential side effects of EVL (8), a cohort of 51 patients who changed to EVL between January 2005 and June 2006 received low doses (0.5 mg bid; designated LD group) to maintain blood levels to 3–5 ug/l. After conversion to EVL, the CSA or TAC dose was reduced gradually in both groups: on the first day of converting to EVL, for safety reasons CSA or TAC dose was reduced only by 20%. During the next 4 days, the CSA or TAC dose was further reduced to achieve an overall reduction of 40%. Purine inhibitors and methylprednisone were stopped after changing patients to EVL. All procedures of this investigation were in accordance with the Helsinki declaration of 1975.

### 2.2. Data Assessment

In the RD and LD groups, we assessed patients' data for up to one year after converting to EVL. The primary endpoint was a composite endpoint of death, graft loss, premature EVL discontinuation due to adverse events, and any event of rejection. Secondary endpoints were safety parameters determined on the basis of laboratory evaluations. In detail, we recorded serum concentrations of triglycerides, total cholesterol, HDL- and LDL-cholesterol, creatinine, blood urea nitrogen (BUN), white blood cells, red blood cells, and platelets before converting to EVL (t0) and at six (t6) and twelve months (t12) after conversion. Blood trough levels of immunosuppressives were recorded before conversion (TAC, CSA), and at t1, t3, t6, t9, and t12 (EVL,TAC,CSA). Immunosuppressives were determined by liquid chromatography tandem mass spectrometry (Waters, Eschborn, Germany). Hematological parameters were analyzed by the Cell-Dyn 3700 (Abbott Diagnostics, Wiesbaden, Germany), whereas the other biochemical parameters were measured by the Architect ci8000 (Abbott, Wiesbaden, Germany). Glomerular filtration rate was assessed by the MDRD formula [11].

### 2.3. Cardiac Rejection

Generally, we do not perform myocardial biopsies routinely after discharge. We assume a clinically proven rejection that needs a daily cortisone bolus of 1000 mg for three days when at least two of the following five criteria are present: an echocardiography assessed ejection fraction <50%, a left ventricular ejection time <200 ms, an isovolumetric relaxation time >60 ms, occurrence of septic hypokinesia and pericardial effusion, and a mean arterial pressure <65 mmHg in parallel with nausea, weakness, and abdominal or thoracic pain. We assume a clinically proven severe rejection which needs OKT therapy when cardiac index is <1.6 L/min/m^2^ (low output syndrome).

### 2.4. Statistics

Categorical variables are reported using the percent of observations. Continuous variables are expressed as mean and standard deviation (SD). For comparisons of categorical variables, the two-sided Fisher exact test or the chi-square were used when appropriate. Comparisons of continuous variables at baseline and at specific time points were performed with the Mann-Whitney test. Time effects were evaluated using the Friedman test. The Wilcoxon test was used to assess differences between groups at specific time points. Complication rates were calculated with the Kaplan-Meier product-limit estimator. The log-rank test was used in order to test for potential differences in complication rates between the study groups. *P* values < 0.05 (two-tailed test) were considered statistically significant. We used the statistical software package PASW, version 18 (Chicago, IL, USA), to perform the analyses. This analysis was designed by the authors, who had full access to the data, analysed the data, and controlled all decisions regarding publication.

## 3. Results and Discussion

### 3.1. Baseline Patients' Characteristics

Baseline characteristics are listed in Table 1. The two groups were comparable with respect to age, sex, body weight and height, primary reason for cardiac transplantation, prevalence of diabetes mellitus, and kidney function. Moreover, the time since cardiac transplantation and the kind of concomitant CNI inhibitor administration did not differ between groups. In the LD group, 33 patients received CSA, whereas 18 patients received TAC. The corresponding numbers for the RD group were 34 and 22 patients, respectively. Mean baseline CSA and TAC levels were within the target range for patients receiving immunosuppression (CSA: 80–100 *μ*g/L;.TAC: 6–10 *μ*g/L). After reduction of the CNI-dose, blood trough levels of CSA and TAC decreased to a similar degree in both study groups (Table 2). As one would expect, blood trough levels of EVL increased less pronounced in the LD group than in the RD group (Table 2). In the LD group, mean EVL concentrations were at the lower end of the target range during follow-up. At t6, mean weekly EVL dose in the LD and RD groups was 9.0 mg (SD: 2.2 mg; median: 8.8 mg) and 13.5 mg (SD: 4.7 mg; median: 12.0 mg), respectively (*P* < 0.001). The corresponding values at t12 were 8.8 mg (SD: 1.6 mg; median: 9.1 mg) and 13.3 mg (SD: 4.4 mg; median: 12.0 mg), respectively (*P* < 0.001). Thus, weekly EVL dose was 50% higher in the RD group than that in the LD group.

### 3.2. Primary Endpoint

**Figure 2:**
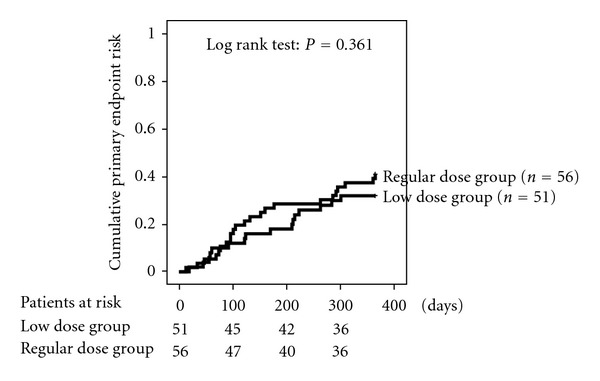
Cumulative risk of the primary endpoint. The primary endpoint was a composite of death, graft loss, everolimus discontinuation and rejection.

Figure 2 illustrates according to study group the incidence of the primary endpoint. In the LD and RD groups, 32.0% and 41.1%, respectively, of patients had reached the primary endpoint (*P* = 0.361). Thus, results did not differ significantly between the two groups. Moreover, the rates of death, premature EVL discontinuation, and episodes of rejection were similar in both groups (Table 3). In detail, there were 2 deaths in the RD group but none in the LD group. Causes of death were noncardiac (traffic accident) and CAV. The number of patients with adverse events leading to discontinuation of EVL was 23.5% in the LD group and 33.9% in the RD group (*P* = 0.166). Eight clinically proven cardiac rejections occurred during follow-up. The prevalence did not differ between the LD and RD group (Table 3). Of the 3 patients with cardiac rejection in the RD group, one patient had EVL blood trough levels below the target range around the time when the event occurred. Of the 5 patients with cardiac rejection in the LD group, four patients had EVL blood trough levels below the target range around the time when the event occurred.

Three years after converting to EVL, the composite endpoint was reached by 45.1% of the patients in the LD group and by 53.6% of the patients in the RD group (*P* = 0.340). In detail, in the LD group 3 patients had died, 1 patient was retransplanted, 5 had developed a cardiac rejection, and 14 had to discontinue EVL. The corresponding values for the RD group were death (*n* = 5), cardiac rejection (*n* = 3), and EVL discontinuation (*n* = 23).

### 3.3. Safety Parameters

In total, there was a trend towards more adverse events in the RD group than in the LD group (*P* = 0.054). Whereas infections were prevalent in the LD group, edema/dyspnoe and cytopenia were the most prevalent adverse events in the RD group (Table 3). A detailed inspection of the adverse events leading to EVL discontinuation also shows that significant more causes of cytopenia resulted in EVL discontinuation in the RD group as in the LD group (Table 3). In contrast, more patients had to discontinue EVL in the LD group than in the RD group due to infections. None of the patients had PCR-proven CMV infection, either in the LD group or in the RD group.

Neither in the LD group nor in the RD group creatinine and BUN concentrations increased during follow-up (Table 4). Lipid parameters and blood cell counts did not differ between the two study groups at any specific point of time. In both study groups, however, trigylcerides and total cholesterol tended to increase during follow-up.

## 4. Discussion

The combination of EVL with a reduction in CNI inhibitors is increasingly used in cardiac transplant recipients [12–14]. There is evidence that this immunosuppressive regimen is able to maintain the low cellular rejection rates seen with standard therapy [4, 15] and may protect kidney function [5, 16]. The present cohort study indicates that low-dose EVL is as effective and safe as regular dose EVL with respect to a composite endpoint of death, rejection, and severe adverse events. In addition, measured creatinine levels showed that kidney function was similar between the two groups at the end of the follow-up period. To the best of our knowledge, this is the first investigation studying the effects of low-dose EVL in cardiac transplant recipients. Results are promising; however, the fact that this is a nonrandomized study precludes us from any final conclusions. Prospective randomized studies are warranted to confirm efficacy and safety of low-dose EVL.

There was significant disparity in the kind of adverse events with more infections but less cases of cytopenia in the LD group than in the RD group. It is well known that mTOR inhibitors such as EVL can result in myelosuppression [9, 10]. This may explain why cytopenia occurred more often in the LD group than in the RD group. However, this is obviously not a general EVL effect in cardiac transplant recipients since mean concentrations of red blood cells, white blood cells, and platelets were unaffected by the introduction of EVL (Table 4). Although none of the patients had CMV infections during follow-up, it may be that several infections were of viral origin in the patients affected. mTOR inhibitors may affect viral amplification indirectly by blocking cellular proliferation and impairing the phosphatidylinositol 3-kinase pathway which is crucial for CMV infection, signalling, and replication [17, 18]. This may at least in part explain the lower infection rate in the RD group compared to the LD group. Our data confirm that many adverse effects under EVL occur early after conversion [19]. While EVL can result in potentially life-threatening adverse events [8], some adverse events resolve without intervention within a few weeks [19]. Nevertheless, the fact that the total number of adverse events tended to be lower in the LD group compared with the RD group supports the use of a low-dose EVL regimen.

The percentage of patients with cardiac rejection was in the range observed in earlier studies in maintenance cardiac transplant recipients receiving EVL [13–15]. Although cardiac rejections did not differ between groups, our data also indicate that the risk of having an event may increase at blood EVL levels below 3 *μ*g/L. Therefore, effective patient adherence and a regular control of EVL blood trough levels are important. Because of the promising results of low-dose EVL administration and as a compromise regarding the risk of cardiac rejection, we are currently using an initial EVL dose of 1.25 mg daily (0.5 and 0.75, resp.). Our target EVL blood level is now 4.0−6.5 *μ*g/L.

Due to the inclusion criteria, the vast majority of our patients had already had poor kidney function when they changed over to EVL. Recent data indicate only a modest improvement in kidney function in patients with already existing renal dysfunction [16, 20]. In line with these earlier findings, our data analysis indicates that neither regular dose EVL nor low-dose EVL resulted in a clinical relevant improvement in kidney function during follow-up, as measured by serum creatinine levels. There is however evidence that EVL treatment has the greatest potential for improving renal function within the first year after cardiac transplantation [14, 21]. Since EVL is less nephrotoxic than CNI inhibitors but still has nephrotoxic potential [22], low-dose EVL may be most effective in this context. However, this has to be investigated in future studies.

Our results of unchanged LDL-cholesterol levels and higher triglyceride levels during follow-up are in line with earlier results [22]. The large standard deviation of triglyceride levels in the RD group indicates that some patients in this group obtained very high levels. Again, some patients seem to be highly responsive to EVL with respect to adverse effect.

Our study has some limitations. Besides the aforementioned fact that it was a nonrandomized investigation, some may criticize that we excluded from our study a large number of patients who were switched over to EVL. However, the vast majority of these patients were at high risk of insufficient immunosuppression due to poor adherence or recurrent rejection. In these patients, it would have been unethical to administer a low EVL dose. Instead, all 56 patients with regular EVL dose who were included in our data analysis would have had the theoretical chance to receive the low EVL dose if they would have been switched over at another point of time. Some may also argue that the follow-up was restricted to one year only. However, even at 3 years of follow-up, the two groups did not differ with respect to major clinical results.

## 5. Conclusions

The results of our cohort study indicate that low-dose EVL may be as effective and safe as regular dose EVL.

## Figures and Tables

**Table 1:** Characteristics of the patients.

Characteristic	1.0 mg of Everolimus (*N* = 51)	1.5 mg of Everolimus (*N* = 56)	*P* value
Age (years)	61.9 ± 10.6	59.0 ± 11.5	0.170
Sex (% males)	84.3	82.1	0.801
Weight (kg)	81.1 ± 12.1	79.6 ± 12.0	0.533
Height (cm)	176 ± 7	174 ± 8	0.165
Primary reason for transplantation			
Dilated cardiomyopathy (%)	43.1	30.4	0.228
Coronary heart disease (%)	47.1	64.3	0.082
Others (%)	9.8	5.4	0.474
Diabetes mellitus (%)	21.6	23.2	0.512
Time since transplantation (months)	76.8 ± 61.8	80.7 ± 60.0	0.675
Glomerular filtration rate (mL/min/1.73 m^2^)	34.4 ± 11.9	35.8 ± 10.3	0.528
Kind of immunosuppression after conversion			
CSA/EVL (%)	66.7	58.9	0.431
TAC/EVL (%)	33.3	41.7	0.431

**Table 2:** Time course of immunosuppressive agents in cardiac transplant recipients receiving low-dose EVL (LD) or high-dose EVL (RD).

Parameter	*T*_0_	*T*_1_	*T*_3_	*T*_6_	*T*_9_	*T*_12_	*P* value
Everolimus (*μ*g/L)							
LD group	—	4.34 ± 2.63	3.59 ± 1.44***	3.66 ± 2.06***	3.34 ± 1.22***	3.10 ± 1.02***	0.139
RD group	—	5.22 ± 2.63	5.76 ± 2.53	6.24 ± 2.49	5.31 ± 1.86	5.28 ± 2.01	0.096
CSA (*μ*g/L)							
LD group	102.0 ± 31.5	70.1 ± 38.8	51.0 ± 15.6	51.4 ± 19.5	47.8 ± 18.8	43.1 ± 13.6	<0.001
RD group	91.3 ± 26.1	60.3 ± 30.0	48.2 ± 27.8	50.6 ± 27.5	48.8 ± 22.4	43.1 ± 19.7	<0.001
TAC (*μ*g/L)							
LD group	9.10 ± 2.10	6.49 ± 2.91	5.26 ± 1.42	5.74 ± 2.36	4.48 ± 0.94	4.75 ± 1.10	<0.001
RD group	8.88 ± 2.14	6.71 ± 2.69	4.89 ± 1.42	4.68 ± 1.28	5.20 ± 2.18	5.67 ± 1.88	<0.001

***Significant different from RD group at the same time point.

**Table 3:** Adverse events of everolimus (EVL) during 12 months of Follow-up^1^.

Adverse event	1.0 mg of EVL (*N* = 51)	1.5 mg of EVL (*N* = 56)	*P* value
Patients who discontinued everolimus treatment before 12 months			
Death	0	2 (3.6)	0.272
Graft loss	0	0	>0.999
Lost to follow-up	0	0	>0.999
Adverse events leading to EVL discontinuation	12 (23.5)	19 (33.9)	0.166
Infection	6 (11.8)	1 (1.8)	0.043
Edema and/or dyspnoe(total)	5 (7.8)	10 (17.9)	0.105
Lingual edema	0	1 (1.8)	0.523
Diarrhea	0	1 (1.8)	0.523
Epitaxis	1 (2.0)	2 (3.6)	0.523
Cytopenia	0	4 (7.1)	0.048
Urticaria	0	1 (1.8)	0.523
All patients	22 (43.1)	35 (62.5)	0.054
Rejection	5 (9.8)	3 (5.4)	0.307
All adverse events			
Infection	10 (19.6)	2 (3.6)	0.009
Edema and/or dyspnoe (total)	6 (11.8)	19 (33.9)	0.011
Lingual edema	0	2 (3.6)	0.225
Diarrhea	0	3 (5.4)	0.105
Epitaxis	1 (2.0)	0	0.523
Cytopenia	0	5 (8.9)	0.022
Urticaria	0	1 (1.8)	0.523

^1^Data are presented as numbers and percentages.

**Table 4:** Time course of biochemical paramters in cardiac transplant recipients on low-dose EVL (LD) or high-dose EVL (RD).

Parameter	*T*_0_	*T*_6_	*T*_12_	*P* value
	LD *n* = 51; RD *n* = 56	LD *n* = 44; RD *n* = 41	LD *n* = 39; RD *n* = 36	
Creatinine (mg/dL)				
LD group	2.23 ± 0.75	2.06 ± 0.87	2.05 ± 0.79	0.030
RD group	2.06 ± 0.44	2.07 ± 0.83	2.08 ± 0.84	0.725
BUN (mg/dL)				
LD group	99 ± 38	81 ± 34	78 ± 29	0.003
RD group	93 ± 37	97 ± 51	90 ± 41	0.420
Triglycerides (mg/dL)				
LD group	162 ± 80	198 ± 106	209 ± 116	0.006
RD group	182 ± 107	254 ± 211	253 ± 222	0.063
Total cholesterol (mg/dL)				
LD group	206 ± 54	216 ± 62	222 ± 51	0.029
RD group	199 ± 50	228 ± 63	229 ± 56	0.013
HDL-cholesterol (mg/dL)				
LD group	57.4 ± 15.7	57.0 ± 19.0	56.0 ± 16.6	0.639
RD group	52.0 ± 15.7	59.4 ± 17.7	59.8 ± 15.4	<0.001
LDL-cholesterol (mg/dL)				
LD group	121 ± 45	125 ± 44	123 ± 41	0.815
RD group	119 ± 33	129 ± 42	124 ± 43	0.611
White blood cells (10^6^/L)				
LD group	7.58 ± 3.40	6.60 ± 1.89	6.36 ± 2.04	0.202
RD group	7.23 ± 1.77	7.20 ± 1.57	7.11 ± 1.78	0.268
Red blood cells (10^12^/L)				
LD group	4.11 ± 0.61	4.32 ± 0.64	4.48 ± 0.74	<0.001
RD group	4.15 ± 0.59	4.35 ± 0.69	4.44 ± 0.73	0.076
Platelets (10^9^/L)				
LD group	218 ± 66	219 ± 65	220 ± 73	0.819
RD group	223 ± 77	224 ± 59	220 ± 52	0.467
